# Education Research: Linguistic Analysis of Racial and Gender Biases in Letters of Recommendation of Neurology Residency Applicants

**DOI:** 10.1212/NE9.0000000000200304

**Published:** 2026-04-14

**Authors:** Ameena Rana, Stefano Malerba, Vicki Lynn Shanker

**Affiliations:** 1 Mount Sinai Hospital Neurology, New York, NY; and; 2 Mount Sinai West, New York, NY.

## Abstract

**Background and Objectives:**

Letters of recommendation (LORs) are a key component of the residency selection process, particularly as objective metrics become less available. Previous studies across specialties have identified implicit gender and racial biases in narrative LORs (NLOR), potentially influencing perceptions of applicants. This study assessed whether such biases are present in NLORs for Neurology residency applicants.

**Methods:**

We conducted a retrospective analysis of NLORs written for all American (US) medical school graduates applying to a single academic Neurology residency program during the 2021–2022 application cycle. Letters were analyzed using Linguistic Inquiry and Word Count (LIWC) software to evaluate structural, cognitive, and emotional language features. Custom dictionaries assessed applicant-related language (e.g., ability, doubt-raising, and gendered terms). Multivariable analyses were performed to examine variations in language use by applicant gender (man vs woman) and race (majority vs underrepresented in medicine [URiM]) across predefined linguistic categories. Analyses were further stratified by letter-writer gender, academic rank, and geographic region.

**Results:**

A total of 431 NLORs were analyzed; 239 (55.8%) were written for female applicants and 72 (16.7%) for URiM applicants. In primary analysis, no significant differences were seen in LORs based on applicant race or gender. Letters for male applicants written by professors showed more gendered language compared with those for female applicants (0.32, IQR: 0.27–0.37 vs 0.15, IQR: 0.13–0.18, *p* = 0.002). Secondary subgroup and regional analyses did not reveal any significant findings.

**Discussion:**

In this linguistic analysis of Neurology NLORs, we did not identify broad or systematic bias in letters written by adult neurologists for Neurology applicants. Our findings suggest that the continued use of NLORs for Neurology residency applicants is appropriate. Larger, multi-institutional studies are warranted to validate these findings and clarify the potential biasing effect of gendered language in LORs.

## Introduction

Letters of recommendation (LORs) remain a critical component of the residency selection process, influencing both interview invitations and final rank decisions.^1-4^ According to the 2021 National Residency Match Program Program Director Survey, the same year the narrative letters analyzed in this study were written, 93.1% of Neurology program directors reported that LORs influenced interview decisions, with an average importance rating of 4.2 out of 5.^5^ Similarly, 75.9% considered LORs when finalizing rank lists (mean rating: 4.0/5), underscoring their ongoing value in applicant assessment.^5^ As residency programs face increasing limitations in objective data due to the USMLE Step 1 transition to pass/fail and the elimination of class rank, AOA designation, and preclinical grades, LORs are likely to carry even greater weight in the selection process.

In response to growing concerns about subjectivity and bias in narrative LORs (NLORs), several specialties including emergency medicine, dermatology, otolaryngology, orthopaedic surgery, and neurosurgery have adopted standardized letter formats (SLORs) to promote greater equity and comparability.^6-9^ This shift has been driven in part by studies demonstrating that NLORs often contain implicit gender and racial biases. Such studies have found that letters for female and URiM applicants often focus on their helpfulness or compassion, whereas letters for male and non-URiM applicants more often highlight confidence, drive, and leadership potential.^10-18^ These linguistic disparities may perpetuate inequities in how competence and readiness are perceived in competitive selection contexts ultimately affecting match outcomes.^10-12,15,17^

Neurology remains one of the few specialties that predominantly continues to use NLORs, yet the presence of gender and race-based linguistic bias within Neurology letters has not been systematically studied. Given the field's reliance on NLORs, it is essential to understand whether similar patterns of implicit bias are present. To achieve this, we examined NLORs written by neurologists for residency applicants, assessing for the presence of gendered and racial linguistic differences using validated linguistic analysis tools to test whether linguistic patterns indicative of bias, as reported in other specialties, were present in Neurology NLORs. We then explored whether letter-writer characteristics such as gender, academic rank, and geographic location influence these patterns. Previous research suggests that such letter-writer characteristics may meaningfully shape letter content and have been explored in similar studies on LORs.^10,14-20^ We hypothesized that linguistic differences by applicant gender or race, similar to those described in other specialties, would be present in Neurology NLORs.

## Methods

### Design

LORs written by neurologists and submitted to a Neurology residency program at a single academic medical center in New York City during the 2021–2022 application year were included in the analysis (n = 431). Letters written by pediatric neurologists were excluded. Letters written only by adult neurologists were included to eliminate any potential confounding factors introduced by the writing style or content of physicians from other fields. Permission from ERAS was obtained, and the study was approved by the Institutional Review Board (IRB) of the organization, Mount Sinai IRB STUDY-24-00367. American (US only) medical school graduates (AMG), MD and DO, were included in the study. Applicant demographic information (self-reported gender and race) was abstracted from ERAS applications.

Letters for applicants were racially grouped into those that are well represented in medicine, Caucasians and Asians (n = 359), and underrepresented minorities (URiMs): Black, Hispanic, and Native American (American Indian, Alaska Native, and Native Hawaiian) (n = 72) based on the classification by the Association of American Medical Colleges.^21^ Any letters associated with applicants missing gender or race self-identification were excluded from the study. Any applicant that self-identified as both Caucasian/Asian and part of an URiM was also excluded to prevent confounding results.

Letters for applicants were grouped by gender as self-identified male (n = 192) or female (n = 239) applicants. Applicants with no self-identified gender were excluded from the study.

Further stratification of data by letter-writer gender, academic rank, and location was chosen as these are factors that could influence the letter content. Letter writers' academic rank (assistant professor, associate professor, professor) and gender were obtained from their letters and, if necessary for clarification, a web search was performed to confirm how the letter writer identified. When letter-writer gender was not explicitly stated, it was inferred using publicly available sources such as institutional websites, professional profiles, and name-based searches, following the methodology of other studies, who used this approach in their analysis of letters of recommendation.^14^ If no pronouns were identified, the gender of the writer was categorized as unknown.

Letter writers were also organized by region of the United States where they were based at the time the letter was written. The regions were grouped as Northeast (Connecticut, Maine, Massachusetts, New Hampshire, New Jersey, New York, Pennsylvania, Rhode Island, and Vermont), Southeast (Delaware, Washington DC, Virginia, West Virginia, North Carolina, South Carolina, Georgia, Alabama, Tennessee, Kentucky, Mississippi, Louisiana, Florida), Midwest (Illinois, Indiana, Iowa, Kansas, Michigan, Minnesota, Missouri, Nebraska, North Dakota, Ohio, South Dakota, and Wisconsin), Southwest (Arizona, New Mexico, Oklahoma, Texas), and West (Alaska, California, Colorado, Hawaii, Idaho, Montana, Nevada, Oregon, Utah, Washington, and Wyoming). Letter writers from the US territories were excluded because of small sample size.

### Linguistic Analysis

Our sample of Neurology NLORs (n = 431) were analyzed using Linguistic Inquiry and Word Count (LIWC 2022). This software program is a validated tool used in multiple similar studies analyzing letters of recommendations in other specialties. This tool “reads” provided texts and analyzes the text against a list of words in both author-generated dictionaries fed to the software program and its own built-in algorithms to provide the results as average word frequency per category.^22^ Custom dictionaries were built by the authors for the categories of “ability,” “agentic,” “communality,” “extracurricular,” “grindstone,” “standout,” “character traits,” “doubt raiser,” “gendered,” and “physical appearance,” referencing custom dictionaries used by other similar studies.^10,15,17,18^

The “ability” category includes terms such as *intelligent, gifted, proficient, and capable*, which refer to an applicant's characteristics as it relates to their work.

The “agentic” category includes terms such as *assertive, aggressive, confident, determined, and efficient*, which refer to the way applicants carry themselves.

The “communality” category includes terms such as *cheerful, agreeable, caring, and compassionate*, which refer to an applicant's interpersonal skills.

The “extracurricular” category includes terms such as *teach, research, volunteer, and mentor,* which refer to an applicant's activities outside of work.

The “grindstone” category includes terms such as *tenacious, deliberate, and meticulous*, which refer to work ethic.

The “standout” category includes phrases such as “*among the best,” AOA, gold humanism, and* “*top 5% of students,”* which refer to common words/phrases for distinctions that set applicants in a top group for a letter writer.

The “doubt raiser” category includes phrases/terms such as *average,* “*accepts criticism well,” and* “*shows improvement”* that indicate a lack of confidence in the applicant.

The “gendered” category includes terms such as *gentleman, lady, wife, and husband* that refer the applicant's gender more specifically.

The “physical appearance” category includes terms such as *beautiful, handsome, well dressed, and bright smile* that refer to an applicant's physical appearance.

The score for each custom category represents the frequency of terminology associated with that category appearing in each letter analyzed.

LIWC's built-in algorithm for “analytical thinking,” “clout,” “authentic,” “tone,” and “affect” was used. High “clout” scores correlate with confidence and a sense of expertise, whereas low scores in this category suggest more timid, humble, or anxious text. High “authentic” scores indicate a more personal approach, and low scores indicate a more guarded or detached text. Analytical thinking captures the amount of formal writing and logical reasoning used in the text with lower scores, indicating language that is “more intuitive and personal.” Finally, a high score for “tone” indicates positivity and optimism, and low scores (less than 50) are associated with negative emotions including uncertainty and ambivalence.^22^

### Statistical Analysis

Before hypothesis testing, the normality assumption of continuous variables was assessed using the Kolmogorov-Smirnov test. Variables displaying significant deviations from normality (*p* < 0.05 for all analyses) were considered non-normally distributed, and the Mann-Whitney *U* test, a non-parametric test for independent samples, was used to examine differences between groups. For normally distributed data, generalized linear models were used. To account for the multiple comparisons made in our analysis, in addition to the standard level of significance of 0.05, we applied the Bonferroni correction, which adjusts the threshold for statistical significance to reduce the likelihood of type I error.^5^ Given that 16 separate tests were conducted, the standard alpha level of 0.05 was divided by 16, resulting in a more conservative significance threshold of 0.003. Statistical analyses were conducted using SAS version 9.4.

The STROBE cross-sectional reporting guidelines were used.

### Standard Protocol Approvals, Registrations, and Patient Consents

The Mount Sinai IRB waived the need for informed consent for the purposes of this study.

### Data Availability

Anonymized data not published within this article will be made available by request from any qualified investigator.

## Results

The distribution of gender and URiM status in our sample closely aligned with the national cohort of 2022 AMGs applying to Neurology residency programs, as provided in Table 1.

**Table 1:** Comparison of Gender and Racial Demographics Between the Study Sample and 2022 National Neurology Residency Applicant Cohort (US Medical School Graduates)

	Data sample	National sample^23^^a^
Applicant gender (%)		
Men	192 (44.54)	309 (47.61)
Women	239 (55.45)	340 (52.39)
Applicant race (%)		
Majority	359 (83.29)	560 (86.69)
URiM	72 (16.71)	86 (13.31)

a National sample data represented here are only inclusive of US medical school graduate applicants' statistics.

### Race-Based Findings

Four hundred thirty-one NLORs met inclusion criteria, comprising 359 letters for applicants from the majority and 72 from URiM racial groups. No significant differences or trends were noted in the primary analysis. Results are summarized in eTable 1.

Female letter writers authored 142 letters (113 for majority applicants and 29 for URiM applicants), whereas male writers authored 289 (246 for majority and 43 for URiM applicants). Results are listed in eTable 2.

A total of 352 letters were written by faculty with an academic rank. Among these, assistant professors authored 144 letters (119 for majority, 25 for URiM applicants), associate professors wrote 105 (91 for majority, 14 for URiM applicants), and professors wrote 103 (86 for majority, 17 for URiM applicants).

Regional subgroup analysis included 418 letters after excluding those for which geographic data were missing or outside the 5 predefined regions (Northeast, Southeast, Midwest, Southwest, and West). No significant difference was seen. Results are provided in eTables 3 and 4.

### Gender-Based Findings

This study included 192 letters for self-identified male applicants and 239 letters for self-identified female applicants. No significant differences were seen among the gender groups in primary analysis. Results are provided in eTable 5.

When stratified by the gender of the letter writer, female letter writers wrote 86 letters for female applicants and 56 letters for male applicants, whereas male letter writers wrote 153 letters for female applicants and 136 for male applicants. No significant differences or trends were found.

Analysis by academic rank included 352 letters authored by academic rank holders. Professors wrote 43 letters for male applicants and 60 for female applicants; associate professors, 53 and 52, respectively; and assistant professors, 57 and 87, respectively. Professors' letters contained significantly more gendered language when written for male applicants (0.32, IQR: 0.27–0.37 vs 0.15, IQR: 0.13–0.18, *p* = 0.002), as provided in Table 2.

**Table 2:** Linguistic Variables^a^ by Applicant Gender (Men vs Women) Stratified by Letter-Writer Academic Rank

	Assistant professor			Associate professor			Professor		
	Women (n = 87)	Men (n = 57)		Women (n = 52)	Men (n = 53)		Women (n = 60)	Men (n = 43)	
	Mean (IQR)	Mean (IQR)	*p* Value	Mean (IQR)	Mean (IQR)	*p* Value	Mean (IQR)	Mean (IQR)	*p* Value
Word count	457.79 (180.0–642.0)	501.54 (186.0–697.0)	0.29	500.90 (139.0–721.0)	558.23 (430.0–690.0)	0.55	583.83 (444.0–751.0)	530.88 (184.0–767.0)	0.41
Ability	58.62 (28.0–77.0)	58.28 (39.0–75.0)	0.83	60.19 (28.0–78.0)	67.49 (44.0–84.0)	0.32	59.75 (39.0–79.0)	55.90 (28.0–92.0)	0.34
Agentic	1.05 (0.74–1.20)	0.88 (0.72–1.09)	0.10	0.93 (0.72–1.13)	1.05 (0.74–1.28)	0.10	0.91 (0.63–1.11)	0.98 (0.68–1.12)	0.24
Communality	0.82 (0.44–1.03)	0.86 (0.48–1.17)	0.66	0.99 (0.42–1.40)	0.93 (0.49–1.41)	0.57	0.67 (0.39–0.94)	0.78 (0.47–0.92)	0.12
Extracurriculars	1.62 (0.99–2.12)	1.77 (1.21–2.28)	0.43	1.96 (1.24–2.68)	1.92 (1.46–2.34)	0.77	2.03 (1.70–2.44)	2.25 (1.61–2.82)	0.38
Grindstone	0.58 (0.34–0.83)	0.46 (0.28–0.61)	0.08	0.42 (0.20–0.54)	0.43 (0.26–0.54)	0.70	0.52 (0.26–0.71)	0.41 (0.19–0.52)	0.32
Standout	0.60 (0.36–0.87)	0.59 (0.36–0.78)	0.54	0.70 (0.42–0.86)	0.65 (0.35–0.83)	0.35	0.63 (0.38–0.80)	0.61 (0.34–0.75)	0.71
Character traits	0.38 (0.19–0.44)	0.41 (0.24–0.56)	0.48	0.38 (0.19–0.51)	0.43 (0.30–0.58)	0.10	0.39 (0.15–0.63)	0.38 (0.28–0.42)	0.79
Doubt raiser	0.18 (0.13–0.26)	0.30 (0.23–0.41)	0.13	—	0.10 (-)		0.16 (0.12–0.16)	0.22 (0.11–0.32)	0.85
Gendered	0.30 (0.16–0.33)	0.41 (0.17–0.55)	0.58	0.19 (0.14–0.25)	0.24 (0.16–0.32)	0.34	0.15 (0.13–0.18)	0.32 (0.27–0.37)	**0.002**
Physical appearance	0.39 (-)	—		0.17 (0.14–0.20)	—		—	0.16 (0.14–0.18)	
Analytic	84.49 (80.28–89.52)	85.45 (80.91–91.20)	0.47	85.96 (82.10–89.52)	85.22 (81.43–90.98)	0.85	86.32 (82.92–90.49)	86.07 (82.08–90.99)	0.99
Clout	59.20 (48.14–73.37)	58.52 (48.57–71.59)	0.68	58.76 (48.61–76.01)	58.65 (46.03–73.90)	0.86	63.80 (58.83–73.60)	58.95 (32.05–75.74)	0.47
Authentic	6.52 (2.69–7.76)	7.76 (2.63–9.58)	0.38	5.22 (2.58–6.20)	7.53 (3.47–9.69)	0.02	5.88 (3.13–7.53)	5.81 (4.00–7.34)	0.93
Tone	62.96 (20.23–85.07)	58.74 (20.23–80.24)	0.14	56.17 (20.23–79.89)	62.29 (43.33–81.32)	0.24	64.00 (53.60–82.64)	58.65 (20.23–79.47)	0.31
Affect	4.21 (3.62–4.89)	3.85 (3.15–4.48)	0.23	3.71 (3.03–4.51)	4.13 (3.64–4.67)	0.32	3.92 (3.23–4.52)	4.05 (3.6–4.49)	0.59

LIWC's built-in categories, including Analytical Thinking, Clout, Authenticity, Tone, and Affect, are scored from 0 to 100 using a proprietary algorithm, which analyzes the linguistic patterns and compares text with large databases to generate a score, with higher scores indicating more formal, confident, personal, and positive emotional content, respectively.^22^

a Custom linguistic categories were developed based on previous literature and include *ability* (e.g., intelligent and capable), *agentic* (e.g., assertive and confident), *communality* (e.g., caring and agreeable), *extracurricular* (e.g., teach and research), *grindstone* (e.g., meticulous and tenacious), *standout* (e.g., “among the best” and AOA), *doubt raiser* (e.g., “shows improvement” and average), *gendered* (e.g., husband and wife), and *physical appearance* (e.g., well dressed and bright smile). The score for each custom category represents the frequency of terminology associated with that category appearing in each letter analyzed.

When stratified by letter writer's region, no significant difference was found when analyzing against applicant gender.

## Discussion

In this study, we conducted a linguistic analysis of Neurology NLORs to evaluate for potential racial and gender bias using a language processing software program. Aside from professors' increased use of gendered descriptors for men, no variables differed significantly by applicant gender or race. Thus, contrary to our initial hypothesis that Neurology NLORs would reflect the gendered and racialized language patterns reported in other specialties, we found no evidence of broad linguistic bias.

The observed increase in gendered descriptor for men in professors' letters may reflect generational linguistic norms, given that many senior neurologists trained during a period in which such terminology was routine in evaluative writing.^24^ The gender-linked language patterns described in other specialties, favoring agentic or ability-related descriptors for men and communal terms for women, were not observed in our data.^10-19^ Without corroborating evidence of differential tone, content, or evaluative framing, isolated gendered terms do not seem to meaningfully reflect bias in this cohort. Previous studies similarly have not shown that gendered terminology alone alters the evaluative impact of letters of recommendation.

Residency selection in Neurology carries substantial stakes. Neurology NLORs remain one of the most influential components of the application. These results provide reassurance to program directors that the narrative letters they rely on are not substantially shaped by gendered or racialized language patterns documented in other specialties. The absence of overt bias may be a sign of growing awareness among neurologists for inclusive communication.

Our findings add nuance to ongoing debates about standardized vs narrative LOR formats. Despite calls for uniformity through SLORs or SLOEs, evidence from other specialties indicates that standardization alone does not eliminate bias and may reduce the richness of narrative assessment.^8,25-27^ A recent analysis of neurosurgery SLORs identified gender bias in the narrative sections, with female applicants more frequently receiving “letters of minimal assurance” and more commonly being described using teaching-related adjectives compared with male applicants.^8^ Similarly, studies in orthopaedic surgery have demonstrated grade inflation, with most applicants rated within the top 2 deciles, thereby reducing the discriminative value of these tools. Applicants rated below the top tiers were also less likely to match despite comparable qualifications, raising concerns about fairness and transparency.^25-27^ Our analysis suggests that well-written narrative letters, when authored conscientiously, can remain a fair and informative component of Neurology's selection process.

Increasing gender diversity within academic Neurology may partly explain why linguistic bias was not detected in our cohort. Women now comprise a substantial portion of academic Neurology faculty and nearly half of current Neurology residents.^28^ This demographic shift means that both writers and subjects of residency letters increasingly represent a more gender-balanced group compared with many surgical specialties where bias was reported. A more diverse academic workforce may naturally reduce the likelihood that letter-writing practices reflect historically gendered patterns.

There is some specialty-specific guidance in the literature to support equitable NLORs. In the study “Pearls and Pitfalls in Letters of Recommendation in Neurology Residency Applications,” the authors provided explicit recommendations for avoiding biased or overly personal descriptors, emphasizing competency-based evaluation and recognizing the potential impact of implicit bias.^29^ The availability and dissemination of such guidance within Neurology may encourage more deliberate and balanced letter writing, reducing variation that might otherwise produce linguistic disparities.

The institutional commitment of the American Academy of Neurology (AAN) to diversity, equity, and inclusion may also contribute to a cultural shift where letter writers are more conscious of bias and more deliberate in their use of language. The Academy offers substantive educational resources such as eLearning modules and webinars on inclusion, antiracism, and equitable practice settings.^30^ Through this education and AAN initiatives to promote underrepresented individuals in careers in Neurology and in leadership, DEI values may now be more embedded in its members. In this environment, letter writers may be more attuned to potential bias and avoid gendered or racialized language.

Future research should build on these findings through larger, multi-institutional, and multiyear analyses to determine whether the absence of broad linguistic bias in this cohort is consistent across regions, program types, and application cycles. Incorporating self-reported demographic data for both applicants and letter writers, including nonbinary and gender-diverse applicants, will be essential for a more comprehensive understanding of identity-linked patterns in evaluative language. Linking linguistic features to objective applicant metrics and match outcomes may clarify whether subtle differences in tone or descriptor strength meaningfully influence selection decisions.

Our finding that professors used more gendered descriptors for men raises the question of whether such language influences program directors' evaluations of these letters. Future research linking letter content to program director ratings or match outcomes could clarify whether these descriptors have meaningful consequences in the selection process.

Finally, future work integrating more context-sensitive language processing tools alongside qualitative analysis will help capture nuance beyond LIWC's categorical structure and may better detect subtle patterns that influence the perceived fairness and narrative richness of NLORs.

Several considerations should be acknowledged when interpreting these findings. This analysis reflects a single application cycle from one urban Neurology residency program and may not capture variations that exist across institutions or regions. However, the demographic composition of our sample closely paralleled national data for the same cycle, supporting its representativeness. Year-to-year variability in applicant and letter-writer demographics could influence linguistic patterns and should be explored in future multiyear analyses.

The gender of letter writers was determined using publicly available data, an approach consistent with previous studies, although self-reported information would provide greater precision. Applicants who did not self-report gender as men or women or did not self-report race, or who identified across majority and minority racial categories, were excluded; this group represented a small proportion of the total sample; however, we recognize the importance of including nonbinary applicants in future studies. Other applicant variables, such as USMLE/COMLEX scores, AOA/GHHS membership, or match outcomes, which may have influenced letter content, were not incorporated in the analysis.

Finally, although LIWC is a validated linguistic analysis tool, it cannot fully account for contextual nuances, for example, treating *proficient* and *exceptional* as equivalent within the “ability” category. We attempted to mitigate this limitation by incorporating additional measures, such as “tone” and “grindstone” attributes, to better capture the qualitative strength of descriptors.

This study presents a linguistic analysis of race and gender-based patterns in Neurology residency NLORs authored by neurologists. In contrast to findings from other medical specialties, our analysis did not identify broad or systematic linguistic bias based on applicant gender or race, suggesting that narrative letters continue to serve as an appropriate component of Neurology's resident selection process.

## Glossary

AMG: American Medical School Graduate
IRB: Institutional Review Board
LIWC: Linguistic Inquiry and Word Count
LORs: letters of recommendation
NLOR: narrative LORs
URiM: underrepresented in medicine
